# Effects of dupilumab on keloid stabilization and prevention

**DOI:** 10.1016/j.jdcr.2023.05.001

**Published:** 2023-05-12

**Authors:** Ashley Wittmer, Lindsey Finklea, Jonathan Joseph

**Affiliations:** a Texas A&M University School of Medicine, Bryan, Texas; b University of Texas Health Science Center at San Antonio, San Antonio, Texas; c The University of Texas at Austin, Austin, Texas

**Keywords:** acne, case reports, dupilumab, general dermatology, keloid, scar

## Introduction

Keloid scarring occurs from many forms of cutaneous injury, including lacerations, burns, surgery, and acne.^1^ Aberrancies in normal physiological responses to injury go unchecked, leading to exuberate fibrous growth and continuous inflammation in the reticular dermis.^1^^,^^2^ It is well-established that ears, cheeks, shoulders, upper arms, upper back, and chest are more prone to keloid scarring.^3^ Keloids have a high rate of recurrence and current therapies have limited efficacy.^2^^,^^4^ Novel approaches have been sought, and dupilumab has recently been described by Diaz et al^5^ to improve keloids in a 53-year-old African American man undergoing dupilumab therapy for atopic dermatitis. Dupilumab is a human monoclonal IgG4 antibody that inhibits interleukin-4 (IL-4) and interleukin-13 (IL-13) signaling by binding to the IL-4Rα receptor subunit affecting cellular transcription.^6^ Diaz et al utilized real-time polymerase chain reaction and found that keloid tissue demonstrated an overexpression of helper T cell type 2 (Th2) genes, particularly interleukin 4 (IL-4) receptor and interleukin 13 (IL-13). Wong and Song^7^ also recently reported a case where dupilumab was used for 3 months in a 37-year-old South Asian woman with a tender, inflamed keloid on the sternum. The authors noted that after this course of dupilumab, her pain and associated symptoms were nearly absent.^7^

In this case report, we discuss 2 cases of severe, chronic keloids where dupilumab was used to stabilize keloid regrowth after surgery and radiation. We also noticed a change in the tissue’s responsiveness to intralesional steroid treatment after initiating dupilumab. Our second patient, with moderate to severe acne, found that adding dupilumab resulted in fewer keloids forming after acne flares.

## Case report

### Patient 1

**Fig 1:**
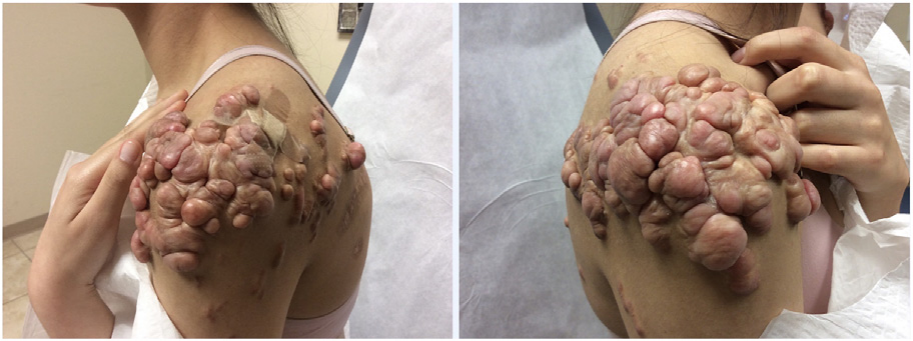
Keloids. Patient’s *left* and *right shoulders* failing multiple attempts of intralesional triamcinolone.

**Fig 2:**
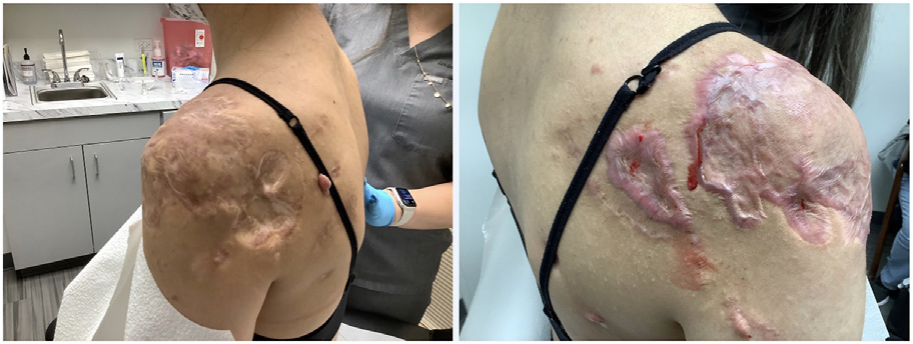
Keloids. Patient’s shoulders following bilateral excision, grafting, and radiation. Patient’s *right shoulder* failing radiation with active inflammation, tenderness, and pruritus.

**Fig 3:**
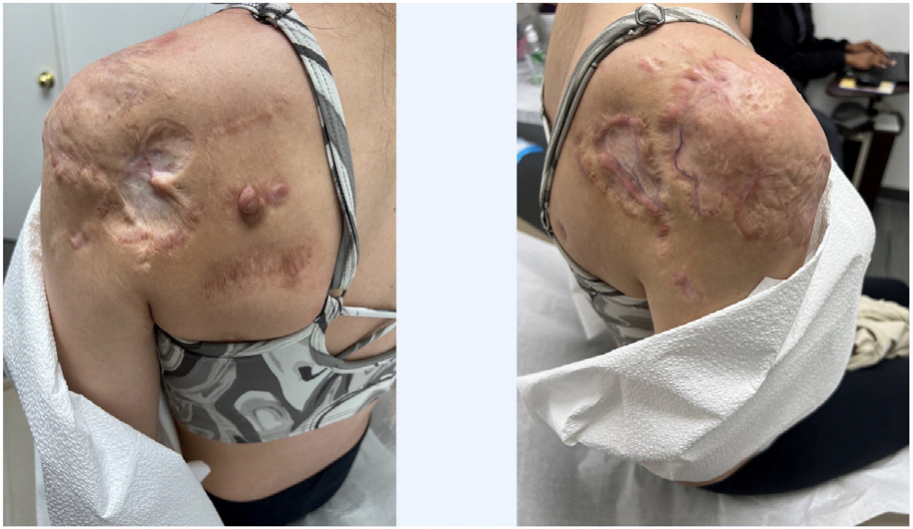
Keloids. Patient’s *left* and *right shoulders* after treatment with dupilumab.

A 23-year-old female presented to our Dermatology clinic in 2018 with tender, disfiguring keloids enveloping both upper shoulders and chest from truncal acne. Her keloids were unsuccessfully managed with intralesional triamcinolone injections for 10 years (Fig 1). At our clinic, she was treated with bilateral excision, followed by skin grafting and 3 fractions of radiation, totaling 18Gy. Her left side remained quiescent, but her right side became tender to palpation and elevated again after 1 year. She reported frequent ulceration and difficulty sleeping due to the itch and discomfort. She was then treated with serial intralesional triamcinolone injections at 40 mg per ml (3 cc total) every 4-6 wk, for a total of 13 treatments. After these treatments, her right shoulder remained inflamed (Fig 2). The patient was then initiated on dupilumab using standard atopic dermatitis dosing, 600 mg of dupilumab subcutaneously, followed by 300 mg every 2 weeks. The pain, pruritus, and inflammation subsided at her 1-month follow-up. After 3 months of stabilization, intralesional therapy resumed with a successful reduction in the size of her keloids. Her right-sided keloid has been quiescent for 8 months (Fig 3). She remains on dupilumab at this time with no plans to discontinue treatment in the near future.

### Patient 2

**Fig 4:**
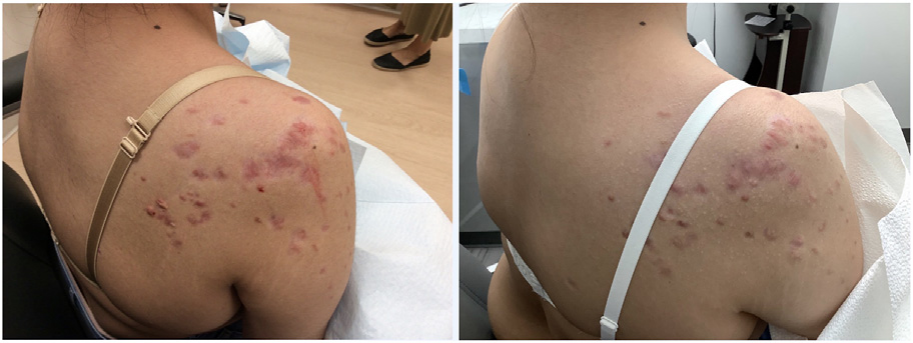
Keloids. Patient’s *right shoulder* after 2 months of dupilumab treatment (*left*) and then after 5 months of dupilumab (*right*).

A 20-year-old female presented to our clinic with several 0.5-1.5 cm keloids on her chest, back, and shoulders, which were all sites of previous acne. She declined isotretinoin but was partially responsive to oral tetracycline class antibiotics. Her periodic flares resulted in additional keloids that were partially responsive to intralesional corticosteroids, but subsequent flares continued to produce new keloids. After initiating dupilumab at 600 mg subcutaneously, followed by 300 mg every 2 weeks, her new acne flares did not result in additional keloids. Two months into treatment, she reported less pruritus and discomfort. Clinically, her lesions appeared less elevated and erythematous. A photo from her 5-month follow-up visit demonstrates further success after additional months of treatment with dupilumab (Fig 4). She is still being treated with dupilumab at this time with no current plans to discontinue.

## Discussion

These cases demonstrate improvement in symptoms of keloids (itch, pain) and allowed our first patient’s keloids to be more amenable to intralesional triamcinolone treatment. Our second patient with ongoing acne was able to prevent new keloids from forming with the addition of dupilumab. To our knowledge, this is the first case where dupilumab was used as an adjunctive therapy to radiation (Patient 1). It is also, according to our understanding, the first reported case of keloid prevention with the use of dupilumab (Patient 2). Patient 1 has been treated for over 11 months with no relapses. This is the longest treatment time with dupilumab for keloids that we are aware of, and patients continue to be monitored at follow ups.

Many questions remain following these cases. As Wong and Song^7^ noted in their case report, the duration of treatment with dupilumab for keloids is yet to be determined. It is uncertain if the atopic dermatitis dosing regimen is optimal for keloids. Perhaps a different dosing schedule would be more appropriate based on keloid severity, patient ethnicity, duration of keloids, or prior radiation.

Interestingly, both patients report that if they are noncompliant with their injections, they quickly begin feeling symptoms of pain and pruritus within a week of missing their dose. This is similar to the sudden onset of pruritus felt in our atopic dermatology population after a missed dose. Further research needs to be conducted to determine the optimal duration of dupilumab and the likelihood of symptoms returning upon discontinuation. Both of our patients are continuing to improve and have no desire to change their injections at this time.

Additional research is also needed to determine who might benefit from Th2 inflammation suppression for keloid prevention. With the initiation of dupilumab, our second patient stopped making new keloids even though her acne periodically flared. If used prior to surgery in high-risk areas or those prone to keloids, could we prevent keloid formation? And, for how long would the dupilumab need to be continued?

Clinical trials are currently being conducted to evaluate the use of dupilumab in reducing keloid size and distensibility. This research protocol will also sample the non-lesional skin of its subjects to begin developing a better understanding of preventing new keloid lesion formation. We encourage researchers to continue seeking an understanding of Th2 inflammation. It is encouraging that new approaches to the unmet need for keloid treatment and prevention are finding new life in this novel pathway.

## Conflicts of interest

Dr Finklea has been a consultant, speaker, or investigator for the following companies: Sanofi, Regeneron, and AbbVie.
